# GABAB receptor inhibits tumor progression and epithelial-mesenchymal transition via the regulation of Hippo/YAP1 pathway in colorectal cancer

**DOI:** 10.7150/ijbs.58135

**Published:** 2021-05-10

**Authors:** Huihui Wang, Hao Zhang, Zhirong Sun, Wankun Chen, Changhong Miao

**Affiliations:** 1 Department of Anesthesiology, Fudan University Shanghai Cancer Center.; 2 Department of Oncology, Shanghai Medical College, Fudan University, Shanghai 200032, China.; 3 Department of Anesthesiology, Zhongshan Hospital, Fudan University; Cancer Center, ZhongShan Hospital, Fudan University; 180# Feng-Lin Road, Shanghai, 200032, China.; 4 Fudan Zhangjiang Institute, Shanghai 201203, China.

**Keywords:** Gamma-Aminobutyric Acid Type B Receptor, proliferation, migration, invasion, epithelial-mesenchymal transition, YAP

## Abstract

Gamma-Aminobutyric Acid Type B Receptor (GABABR) plays essential roles in tumor progression. However, the function of GABABR in colorectal cancer (CRC) needs further clarification. As the main part of GABABR, GABABR1 expression was identified significantly lower in tumor tissues than those in non-tumor normal tissues and that CRC patients with high GABABR1 expression lived longer. Further studies indicated that knockdown of GABABR1 elevated CRC cell proliferation, migration, and invasion. Furthermore, knockdown of GABABR1 activated the expression of the epithelial-mesenchymal transition (EMT)-related proteins N-cadherin and Vimentin, whereas decrease the protein level of E-cadherin. In addition, activation of Hippo/YAP1 signaling contributes to the GABABR1 down-regulation promoted proliferation, migration, invasion and EMT in CRC cells. At last, we verified the contribution of Hippo/YAP1 signaling in the GABABR1 down-regulation impaired biological phenotype of colon cancer cells *in vivo*. In summary, these data indicate that GABABR1 impairs the migration and invasion of CRC cells by inhibiting EMT and the Hippo/YAP1 pathway, suggesting that GABABR1 could be a potential therapeutic target for CRC.

## Introduction

Colorectal cancer (CRC) is the third most common cancer and the fourth leading cause of cancer death worldwide, accounting for almost 900,000 deaths in 2018 1, 2. Surgery has long been established as the primary treatment for CRC. Benefiting from early diagnosis, recent immunotherapy and anti-tumor agents used for treating CRC, the mortality is remarkably reduced; however, the recurrence rates are discouragingly high, and the median overall survival rate for patients remains unsatisfactory. Hence, the core molecular mechanisms underlying CRC progression need further study, and exploring novel therapeutic targets is crucial to improve the survival rates of CRC patients.

Gamma-amino butyric acid (GABA), which acts on ionotropic (GABA_A_ or GABA_C_) and metabotropic (GABA_B_) receptors, is one of the most important inhibitors of neurotransmitters 3. GABA_A_ receptor is pentameric ligand-gated chloride channel, represents the target receptors for major general anesthetics and benzodiazepines. GABA_C_, named GABA-A-rho, is typically classified as a subtype of GABA_A_ 4. GABA_B_ receptor, a G protein-coupled receptor (GPCR), functions as a constitutive heterodimer composed of the GABABR1 and GABABR2 subunits. Research increasingly suggests that the GABA_B_ receptor (GABABR) is involved in tumor progression 5. Previous studies showed that the GABABR antagonist CGP had anti-tumor effects in high-grade chondrosarcoma cells and regulated proliferation of the chondrosarcoma cell line OUMS-27 via apoptotic pathways 6. Another study indicated that the proliferation and migration of ovarian cancer cells were inhibited by down-regulating the mRNA levels of GABAB receptor 7. In addition, GABBR1 induces secretion of the peptide gastrin from neuroendocrine-like cells that are involved in prostate cancer progression 8. Recently, studies have also reported that GABABR regulates the proliferation of colorectal cancer cells by the GSK-3β/NF-κB signaling pathway 9. However, the exact function of GABABR in CRC needs further clarification.

The Hippo pathway is a highly conserved master regulator of organ size and tissue regeneration, and it regulates various biological functions, such as proliferation, apoptosis, viability, and differentiation 10. Dysregulation of the Hippo pathway is associated with multiple pathological disorders, such as a broad range of cancer types, diabetes, and neurodegenerative diseases 11. Many studies have revealed that aberrant activation of YAP is one of the most important mechanisms accounting for the progression of cancer. Hippo/YAP signaling has been reported to regulate proliferation, migration, and invasion in pancreatic cancer cells 12. In gastric carcinoma, Hippo/YAP signaling plays a critical role in epithelial-mesenchymal transition (EMT) 13. Moreover, the phosphorylation of YAP is involved in the regulation of proliferation and apoptosis in hepatocellular carcinoma 14. According to recent reports, through downregulation of LATS1 activates YAP, and may contribute to the progression of CRC 15. However, the exact mechanism remains unclear. Multiple studies have shown that GCPRs are one of many upstream regulators of Hippo/YAP pathway 16, 17. Also, GPCR-Hippo pathway was considered as a potential target for cancer molecular targeting therapy 18. As one of GCPRs, GABABR might be closely associated with YAP.

This study aims to evaluate the role of GABABR in CRC progression. In the present study, we analyzed the relationship between GABABR and the prognosis of CRC patients, the experimental work presented here explored the mechanism of GABABR's role in CRC, which may provide new avenues for CRC research.

## Materials and Methods

### Immunohistochemistry

This study was approved by the Ethics Committee of Fudan University Shanghai Cancer Center (FUSCC), China. Tumor tissues embedded in paraffin were cut into 3 µm sections, followed by deparaffinization and rehydration. Immunohistochemical staining was performed with antibodies to GABABR1 (Abcam, 1:100, Cambridge, UK) and GABABR2 (Abcam, 1:100) at 4 °C overnight. The sections were incubated with peroxidase-conjugated secondary antibodies for 1 h at 37 °C and were mounted in a mounting medium containing glycerol (Beyotime, China). The analysis of positively stained tumor cells was conducted and images were taken under a light microscope.

### Cell culture

RKO, DLD1, Lovo, HCT116, HT29 and SW620 human CRC cell lines were a kind gift from Prof. Changhong Miao (Fudan University Shanghai Cancer Center, China) and were cultured in DMEM (HyClone, Thermo, USA) supplemented with 10% FBS (Biological Industries, State of Israel); cells were incubated at 37 °C in a 5% CO2 incubator.

### Reagents

Anti-GABABR1 and anti-GABABR2 mAbs were purchased from Abcam (Cambridge, UK). N-cadherin, E-cadherin, Vimentin and Hippo/YAP1 pathway antibodies were purchased from CST (Danvers, MA, USA). An anti-actin mAb, an anti-GAPDH mAb and an anti-histone H3 were purchased from Proteintech (Chicago, USA).

### Western blot analysis

Cells were lysed with lysis buffer (Beyotime, China) containing protease inhibitors, PMSF (Beyotime, China). Cellular proteins were separated by 10% SDS- PAGE and were then transferred onto a Hybond TM-P membrane (GE Healthcare, UK). The membrane was blocked with 5% skim milk in TBST for 1 h at room temperature. Then, the membrane was incubated with primary antibodies overnight at 4 °C. Next, the membrane was incubated with peroxidase-conjugated secondary antibodies for 1 h after washing with TBST three times. The blots were then treated with a chemiluminescent reagent (Merck Millipore, USA) and visualized on X-ray films (MidSci, USA). All results were repeated for three times.

### Q-PCR

Total mRNA was isolated from RKO and Lovo cells using Trizol® (Invitrogen, Carlsbad, CA). Then, mRNA was reverse transcribed into cDNA. Real-time reverse transcription polymerase chain reaction (Q-PCR) was performed to detect the expression of N-cadherin, E-cadherin and Vimentin with iTaq™ SYBR Green Supermix (Bio-Rad, CA) in a Step One Plus™ Real-Time PCR System (Life Technologies, USA), according to the manufacturer's instructions (For primer sequences, see Supplementary Table 1). All results were repeated for three times.

### Wound healing assay

Cells were inoculated into a 6-well plate and were cultured to complete confluence. After removing the medium, the cell layers were scraped using a 10 μl pipette tip. After rinsing out the scraped cells, the remaining cells were incubated with serum-free medium for 24 h at 37 °C. The distance of wound closure was recorded to measure the degree of wound healing. All results were repeated for three times.

### Transwell assay

Cell invasion assays were performed in a transwell chamber (24-well, 8 μm pore size; Corning). RKO and Lovo cells were resuspended in serum-free DMEM medium, and 6×10^4^ cells suspended in 200 μl were added to the upper chamber membranes. The bottom was coated with 1 mg/ml BD Matrigel matrix, and 500 μl of complete medium was placed into the lower chamber as a chemoattractant. Then, the cells were incubated at 37 °C in a 5% CO_2_ incubator. After 24 h, the non-invaded cells on the surface of the membrane were removed using a cotton swab. The invaded cells on the lower surface were fixed with 4% paraformaldehyde and stained with 0.5% crystal violet. Each experiment was performed in triplicate. The number of invaded cells was counted in 4 fields for each transwell, and the data were analyzed using ImageJ software. All results were repeated for three times.

### CCK-8 assay

Cells were inoculated into a 96-well plate with 2000 cells/well. The wells without cells were used as the blank control group. Each group had five wells. Cell counting kit-8 (CCK-8) reagent was added, and then the cells were incubated for 2 hours in the dark. The wavelength of the enzyme marker was assessed at 450 nm, and the OD value was measured. All results were repeated for three times.

### Tumor xenografts

Five-week-old BALB/c nude mice were purchased from SLAC Animal Center (Shanghai, China) and then used for xenograft tumor model. The animal experiments were approved by the Institutional Animal Care and Use Committee of the Fudan University. The control and lentivirus mediated stable GABABR1 knock-downed Lovo cells were subcutaneously injected to nude mice and then tumor volumes were monitored every 3 days. The YAP1-TEAD inhibitor Peptide 17 (2 ug/kg) were injected by tail vein every 5 days. Tumor volumes were estimated by length and width and calculated as the following formula:

Tumor volume = (length * width^2)/2

About one month later, the nude mice were sacrificed and then tumors were excised, pictured, and weighed.

### Statistical analysis

Statistical analysis was performed with SPSS 17.0 (SPSS Inc., Chicago, IL, U.S.). Each experiment was repeated at least three times. The results were reported as the mean ± standard deviation (SD). Differences were considered statistically significant at P<0.05.

## Results

### GABABR1 was down-regulated and negatively associated with the adverse clinicopathological features in colorectal cancer

**Figure 1:**
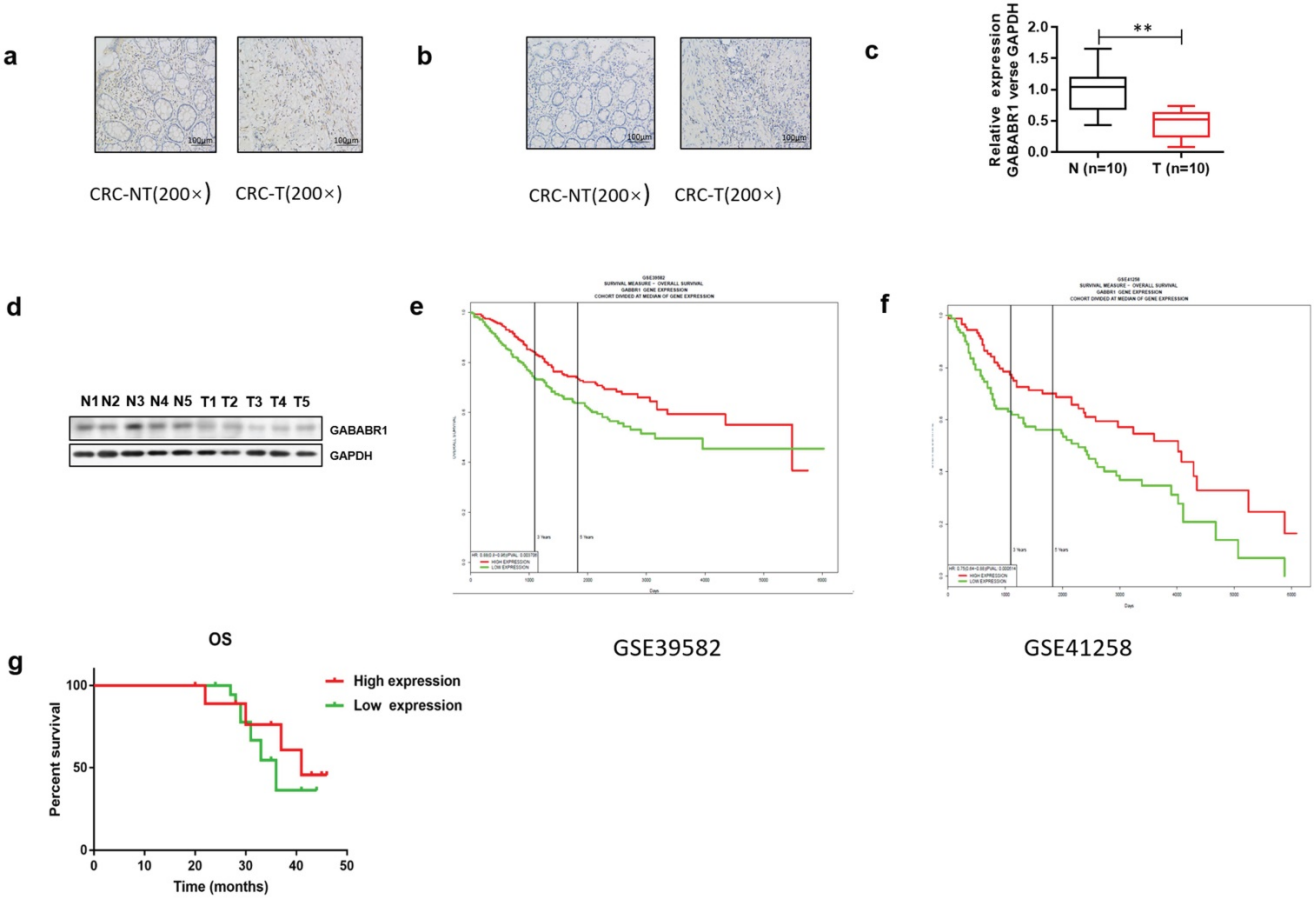
** GABABR1 was down-regulated and negatively associated with the adverse clinicopathological features in colorectal cancer.** (A-B) Representative immunohistochemical staining of GABABR1 and GABABR2 in human CRC tissue (CRC-T) and normal tissues (CRC-NT); scale bar: 100 µM. (C) The mRNA level of GABABR1 in normal adjacent tissues and colon tissues were evaluated by real-time PCR. (D) The protein level of GABABR1 in normal adjacent tissues and colon tissues were evaluated by Western Blot. (E-F) We searched PROGgeneV2 and analyzed the effect of GABABR1 expression on survival of CRC patients. (G) A Kaplan-Meier curve of overall survival in 40 patients is shown with CRC stratified by the expression level of GABABR. The duration of survival was measured from postoperative day 1 until death or 44 months later. *P<0.05 versus indicated group.

To investigate the role of GABABR, we first found that the expression of GABABR1 in tumor tissues was significantly lower than those in normal adjacent tissues from patients with CRC by using immunohistochemistry Compared with the normal adjacent tissues, the rate of GABABR1 positive cells (5%) was decreased in tumor tissues (**Figure 1A**). However, the expression of GABABR2 was too low to detective in both tumor tissues and normal adjacent tissues (**Figure 1B**). Then, we evaluated the mRNA level of GABABR1 and GABABR2 in ten paired normal adjacent tissues and colon tissues. It was found that the mRNA level of GABABR1 in colon tissues was also significantly decreased (**Figure 1C**). We next confirmed the decreased protein expression of GABABR1 in colon tissues by Western Blot (**Figure 1D**. Furthermore, we searched PROGgeneV2 and found two datasets (GSE39582 19 and GSE41258 20). We found that the high expression of GABABR1 was associated with a significant lengthening of survival in CRC patients (**Figure 1E and 1F**). Then, we used 40 cases of CRC specimens surgically removed during January 2015 to June 2015 at Fudan University Shanghai Cancer Center (FUSCC), and analyzed the effect of GABABR1 expression on overall survival (OS). Patients with higher GABABR1 mRNA level than the average for all patients were assigned to GABABR1 high group and patients with lower GABABR1 mRNA level were assigned to GABABR1 low group. The Kaplan-Meier survival curves showed that patients with higher GABABR1 expression also had a significantly better 3-year OS (**Figure 1G**).

### Down-regulation of GABABR1 promotes proliferation, migration, and invasion in CRC cells

**Figure 2:**
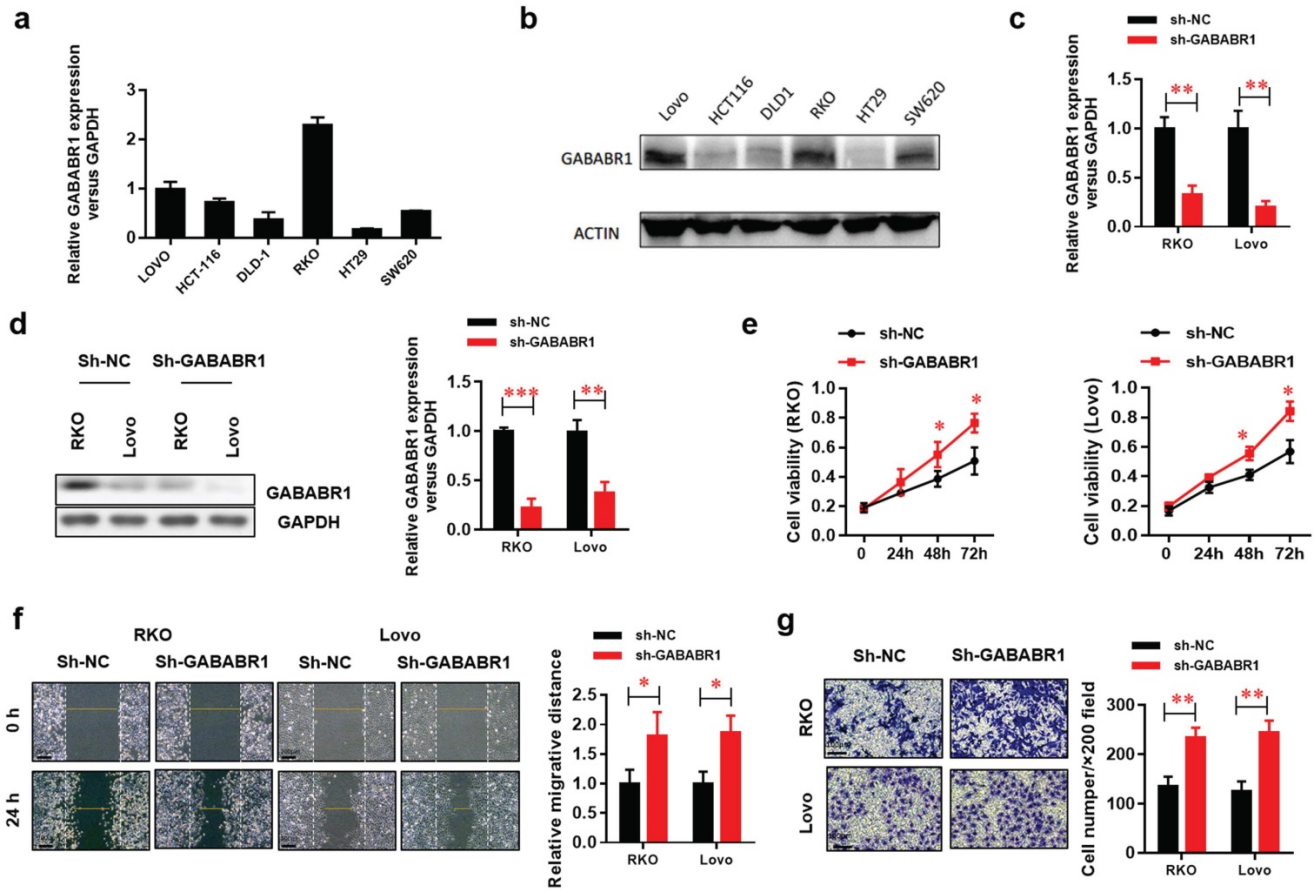
** Down-regulation of GABABR promotes proliferation, migration, and invasion in CRC cells.** (A) Real-time PCR was used to measure the levels of GABABR in six CRC cell lines. (B) GABABR protein levels were tested by western blot. (C) The efficiency of GABABR1 mRNA knockdown was evaluated by real-time PCR. (D) The efficiency of GABABR1 protein knockdown was evaluated by Western Blot. (E) The effect of GABABR1 knockdown on the cell viability of RKO and Lovo cells were evaluated by CCK-8 assay. (F) The effect of GABABR1 knockdown on the migration of RKO and Lovo cells were evaluated by wound healing assay. (G) The effect of GABABR1 knockdown on the invasion of RKO and Lovo cells were evaluated by Matrigel invasion assay. N=3, *P<0.05 versus indicated group.

Based on the results observed in CRC patients, we detected the protein and mRNA expression of GABABR1 in several human CRC cell lines and finally chose two cell lines (RKO and Lovo) with higher expression of GABABR1as experimental models (**Figure 2A and 2B**). The GABABR1 level was knockdown by specific shRNA target to GABABR1 mRNA CDS region. The efficiency of knockdown was evaluated by real-time PCR (**Figure 2C**) and Western Blot (**Figure 2D**). The results showed that GABABR1 knockdown dramatically improved the proliferation (**Figure 2E**), migration (**Figure 2F**), and invasion (**Figure 2G**) abilities, indicated by CCK-8, wound healing and Matrigel invasion assays, respectively. Meanwhile, we found that the migration rate was significantly reduced in RKO and Lovo cells treated with baclofen (an agonist of GABABR, 10 μM) (**Supplementary Figure 1A**). To evaluate the action of GABABR on cell invasion ability, we performed BD Matrigel invasion assays with RKO and Lovo cells and found that activation of GABABR reduced the number of invading cells in both cell types (**Supplementary Figure 1B**). We also assessed the effect of GABABR on the proliferation of CRC cells. We found that activation of GABABR inhibited the proliferation of RKO and Lovo cells (**Supplementary Figure 1C**). These results demonstrated that down-regulation of GABABR promotes proliferation, migration, and invasion in CRC cells. On the other hand, CGP52432, one of the antagonists of GABABR1, consistently enhanced the proliferation (**Supplementary Figure 2A**), migration (**Supplementary Figure 2B**), and invasion (**Supplementary Figure 2C**) abilities, indicated by CCK-8, wound healing and Matrigel invasion assays, respectively. These results suggested that down-regulation of GABABR promotes proliferation, migration, and invasion in CRC cells.

### Down-regulation of GABABR1 activates EMT in CRC cells

**Figure 3:**
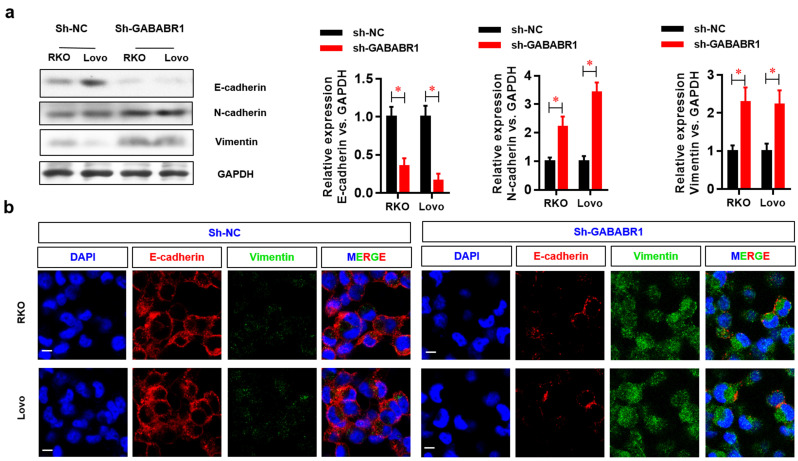
**Down-regulation of GABABR1 activates EMT in CRC cells.** (A) The effect of GABABR1 knockdown on the protein level of EMT related genes, including E-cadherin, N-cadherin and Vimentin were detected by Western Blot. (B) The dysregulated expression of E-cadherin and vimentin were visualized by immunostaining and confocal imaging. N=3, *P<0.05 versus indicated group.

EMT plays a critical role in tumor migration and invasion 21. In this study, the protein expression levels of the EMT-related genes N-cadherin and Vimentin were evaluated by Western Blot and found to be elevated, whereas the protein level of E-cadherin was decreased by GABABR1 knockdown (**Figure 3A**). Moreover, the EMT morphology changes were confirmed, indicating by dysregulated expression of E-cadherin and vimentin were visualized by immunostaining and confocal imaging (**Figure 3B**).

### Activation of Hippo/YAP1 signaling contributes to the GABABR1 down-regulation promoted proliferation, migration, and invasion in CRC cells

**Figure 4:**
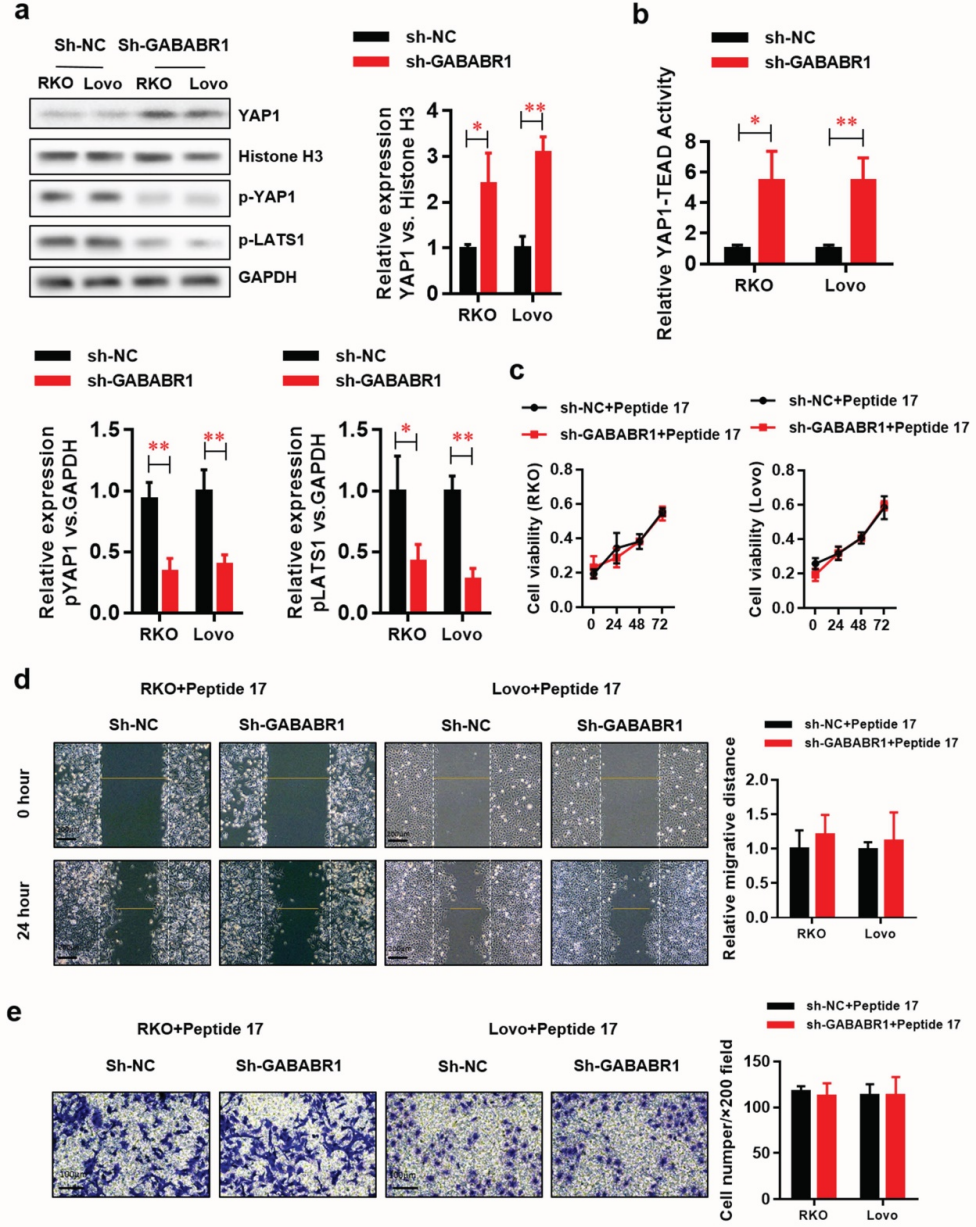
** Activation of Hippo/YAP1 signaling contributes to the GABABR1 down-regulation promoted proliferation, migration, and invasion in CRC cells.** (A) The effect of GABABR1 knockdown on the protein level of Hippo-signaling pathway, including nucleus YAP1, phosphorylated YAP1 and phosphorylated LATS1 were detected by Western Blot. (B) The effect of GABABR1 knockdown on the activity of Hippo-signaling pathway were detected by luciferase assay. (C) The effect of Peptide 17 (1 µM) treatment on the cell viability of GABABR1 knockdown RKO and Lovo cells were evaluated by CCK-8 assay. (F) The effect of Peptide 17 treatment on the migration of GABABR1 knockdown RKO and Lovo cells were evaluated by wound healing assay. (G) The effect of Peptide 17 treatment on the invasion of GABABR1 knockdown RKO and Lovo cells were evaluated by Matrigel invasion assay. N=3, *P<0.05 versus indicated group.

Studies suggest that the Hippo/YAP pathway is involved in EMT 22. The datasets from PROGgeneV2 prompt that GABABR may be involved in the regulation of Hippo/YAP pathway. Herein, we found that protein level of functional type YAP1 was elevated in the nucleus fraction of GABABR1 knockdown RKO and Lovo cells (**Figure 4A**). Meanwhile, the non-functional type YAP1 (phosphorylated YAP1) and its upstream phosphorylated protein LATS1 was decreased. Consistently, the luciferase reporter assay showed that the YAP1-TEAD activity was significantly elevated by GABABR1 knockdown in RKO and Lovo cells (**Figure 4B**). Interestingly, Peptide 17, a specific inhibitor of YAP-TEAD, made an almost complete reverse on the GABABR1 knockdown elevated proliferation (**Figure 4C**), migration (**Figure 4D**), and invasion (**Figure 4E**) abilities, indicated by CCK-8, wound healing and Matrigel invasion assays, respectively.

### Activation of Hippo/YAP1 signaling contributes to the GABABR1 down-regulation activated EMT in CRC cells

**Figure 5:**
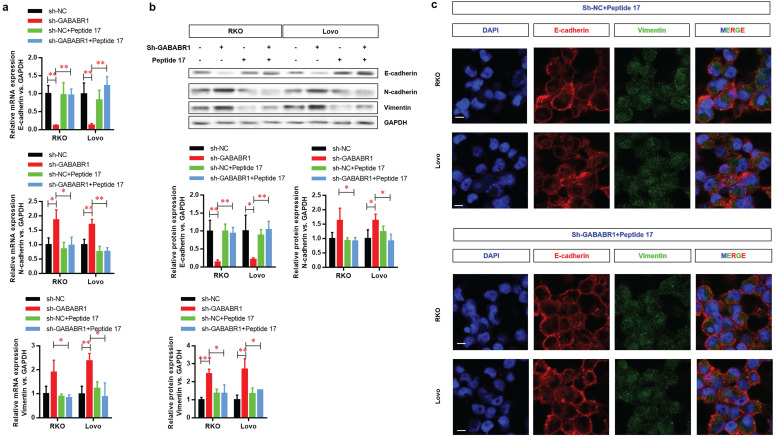
** Activation of Hippo/YAP1 signaling contributes to the GABABR1 down-regulation activated EMT in CRC cells.** (A) The effect of Peptide 17 treatment on the mRNA level of EMT related genes, including E-cadherin, N-cadherin and Vimentin were detected by Western Blot. (B) The effect of Peptide 17 treatment on the protein level of EMT related genes, including E-cadherin, N-cadherin and Vimentin were detected by Western Blot. (C) The dysregulated expression of E-cadherin and vimentin were visualized by immunostaining and confocal imaging. N=3, *P<0.05 versus indicated group.

To further explore whether Activation of Hippo/YAP1 signaling contributes to the GABABR1 down-regulation activated EMT in CRC cells, we then evaluated the mRNAs (**Figure 5A**) and protein (**Figure 5B**) levels of EMT-related genes, including E-cadherin, N-cadherin, and Vimentin. The results showed that the GABABR1 knockdown decreased E-cadherin and increased N-cadherin and Vimentin were also reversed to comparable level with control group. Furthermore, the EMT morphology changes were also confirmed, indicating by reversed expression of E-cadherin and vimentin were visualized by immunostaining and confocal imaging (**Figure 5C**).

### Activation of Hippo/YAP1 signaling contributes to the GABABR1 down-regulation increased tumor growth *in vivo*

**Figure 6:**
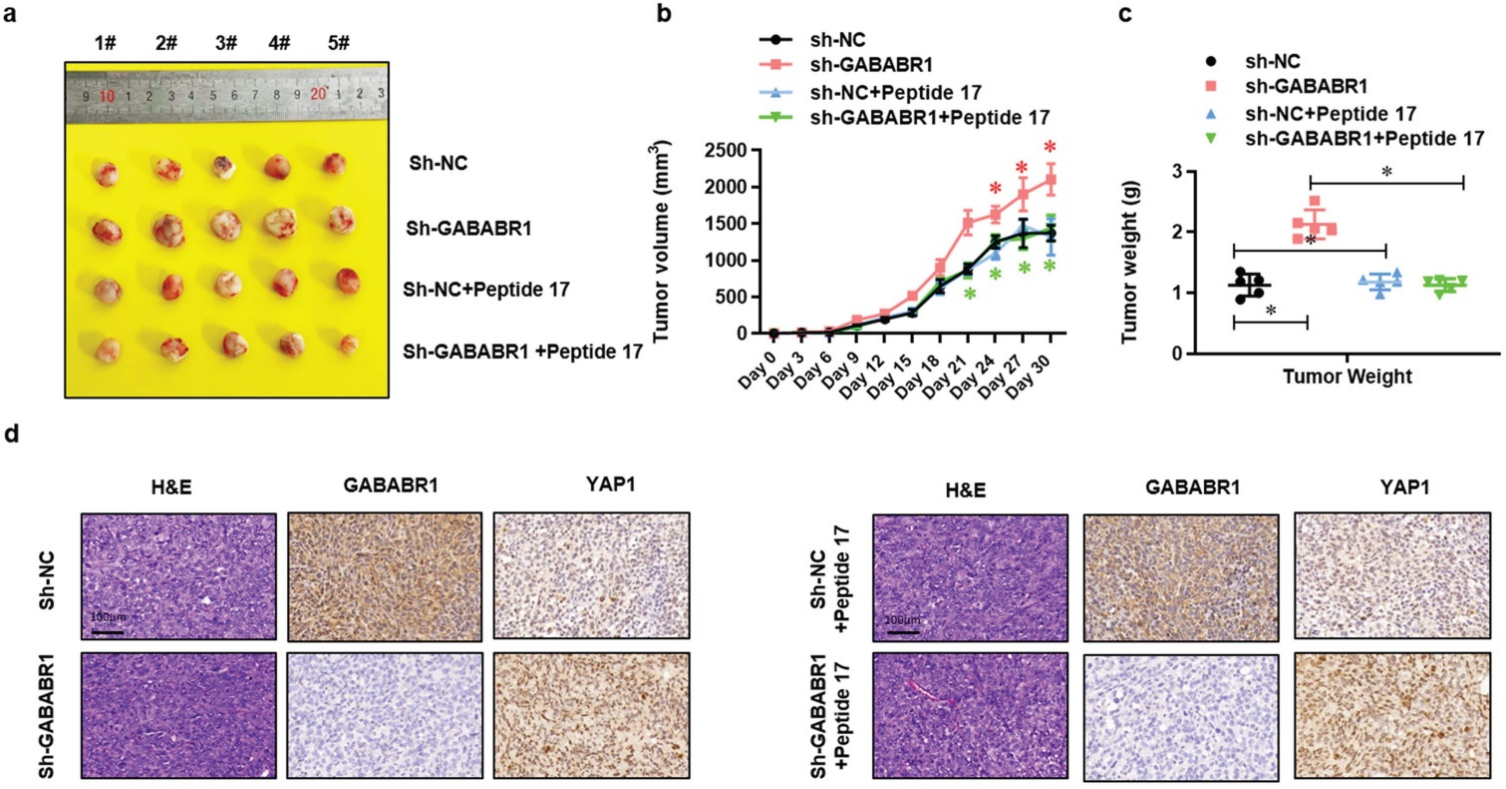
** Activation of Hippo/YAP1 signaling contributes to the GABABR1 down-regulation increased tumor growth *in vivo*.** (A) Representative photographs of the tumor-xenografted nude mice. (B) The tumor growth curve of the nude mice bearing Lovo cells with or without GABABR1 knockdown or Peptide 17 treatment. (C) The tumor weight of the nude mice bearing Lovo cells with or without GABABR1 knockdown or Peptide 17 treatment. (D) H&E, GABABR1 and YAP1 staining of subcutaneous tumors derived from Lovo cells with or without GABABR1 knockdown or Peptide 17 treatment. Scale bars: 100 μM. N=5, *P<0.05 versus indicated group.

To further evaluated the role of Hippo/YAP1 signaling in the GABABR1 down-regulation impaired biological phenotype of colon cancer cells *in vivo*, we constructed a colon cancer Xenograft mice model via subcutaneous injection of normal or GABABR1 knockdown Lovo cells with or without Peptide 17 treatment. Xenograft assays showed that GABABR1 knockdown dramatically increased tumor growth compared with the control group (**Figure 6A**), while YAP1-TEAD inhibition with Peptide 17 reversed tumor volume (**Figure 6B**) and weight (**Figure 6C**) regardless of the GABABR1 knockdown. H&E staining and IHC staining confirmed the expression levels of GABABR1 and YAP1 in these tumors (**Figure 6D**). These results collectively demonstrated that activation of Hippo/YAP1 signaling contributes to the GABABR1 down-regulation increased tumor growth *in vivo*.

## Discussion

CRC has been challenging to treat because it tends to recur frequently. However, the molecular and cellular mechanisms of CRC are not entirely known. GABABR was verified to be associated with the progression of various tumors. This study showed that GABABR1 inhibited the Hippo/YAP1 pathway, suppressed the expression of EMT- related protein, and finally alleviated the migration and invasion of CRC.

As an inhibiting neurotransmitter, previous prospective studies on GABABR mainly involved neurons, neuropathic pain and neuroinflammation 23-25. The role of GABABR in cancer has received increasing attention in recent years. Studies have shown that GABABR is closely associated with tumor cell apoptosis in cancers such as breast cancer, renal carcinoma, and gastric cancer 5, 26. Nevertheless, its exact mechanism has not been illustrated thus far. In this study, we first found that the prolonged OS was associated with higher expression of GABABR1, suggesting that patients with lower expression of GABABR1 may have a poor prognosis.

Metastatic recurrence is the major obstacle preventing improvement of the prognosis for CRC patients. In investigating the function of GABABR1 in CRC progression, we first found that activation of GABABR1 attenuated the proliferation of CRC cells. This was consistent with previous research by Shu et al. 9. Then, we tested the migration rate and number of invading cells in RKO and Lovo cells treated with baclofen. Our results showed that activation of GABABR1 alleviated CRC cell migration and invasion. To test this further, we used CGP52432 or shRNA to inhibit the expression of GABABR1. Contrary to previous results, downregulation of GABABR1 promoted CRC cell migration and invasion. These results helped us confirm that GABABR1 inhibits migration and invasion of CRC.

To understand how GABABR1 regulates CRC migration and invasion, we further analyzed the datasets from PROGgeneV2 and found that GABABR1 may be involved in the regulation of Hippo/YAP pathway. It is well known that YAP plays a role in promoting tumorigenesis, cell proliferation, and resistance to apoptosis in tumor cells 10. Here, we examined YAP1 and the phosphorylation of YAP1 and LATS1 and YAP-TEAD activity. We found that downregulation of GABABR1 promoted activation of the Hippo/YAP1 pathway in GABABR1 knockdown RKO and Lovo cells. However, treatment with Peptide 17 made an almost complete reverse on the GABABR1 knockdown elevated proliferation, migration and invasion abilities, respectively. This indicated that GABABR1 probably regulated EMT through Hippo/YAP pathway. Recently, research has indicated that the YAP pathway is involved in the regulation of EMT and metastasis 27, 28. EMT, an essential regulatory process that mediates invasion and metastasis, is considered a culprit of metastasis in CRC 29. There are many pathways upstream of EMT, such as Wnt/β-catenin, JNK, and TGF-β 25, 26. In this study, the expression of EMT-related molecules N-cadherin and Vimentin were found to be elevated, and E-cadherin was suppressed (at the protein and mRNA level) in GABABR1 knockdown RKO and Lovo cells. Then, this trend partly reversed with piptide 17 treatment. Therefore, GABABR1 might inhibit the EMT process to regulate CRC migration and invasion by Hippo/YAP1 pathway. To further investigate the role of GABABR1 and Hippo/YAP pathway, we constructed a colon cancer Xenograft mice model. The results showed that activation of Hippo/YAP1 signaling contributes to the GABABR1 down-regulation increased tumor growth *in vivo*. These results revealed that the Hippo/YAP pathway might be upstream of EMT and that GABABR inhibits migration and invasion via the Hippo/YAP pathway and EMT.

Taken together, these findings indicate that GABABR1 exhibits an anti-tumor effect. The aberrant expression of GABABR1 regulates the EMT to suppress migration and invasion through the Hippo/YAP1 pathway, implicating GABABR1 as a therapeutic target in CRC.

## Supplementary Material

Supplementary figures and tables.

## Abbreviations

GABABR: Gamma-Aminobutyric Acid Type B Receptor
YAP: Yes-associated protein
LATS1: large tumor suppressors 1
