# Supplementary material for: GABAB receptor inhibits tumor progression and epithelial-mesenchymal transition via the regulation of Hippo/YAP1 pathway in colorectal cancer
Source: Int J Biol Sci. 2021 May 10;17(8):1953–62. doi: 10.7150/ijbs.58135 (PMC8193267; doi:10.7150/ijbs.58135)
Supplement: Supplementary file 1 — Supplementary figures and tables. [file ijbsv17p1953s1.pdf]

Supplementary Table S1

Gene	Forward primer	Reverse primer
**GABABR1**	5′ - gaggacgtgaatagccgcag-3′	5′ - ctggatcacacttgctgtcgt-3′
**GABABR2**	5′ - ccgcaacgagtcactcctg-3′	5′ - caagtggttaggcccgtatttta-3′
**E-cadherin**	5′ - tggaggaattcttgctttgc-3′	5′ - cgtacatgtcagccagcttc-3′
**N-cadherin**	5′ - tcaggcgtctgtagaggctt-3′	5′ - atgcacatccttcgataagactg-3′
**GAPDH**	5′ - gcaccgtcaaggctgagaac-3′	5′ - ccacttgattttggagggatct-3′

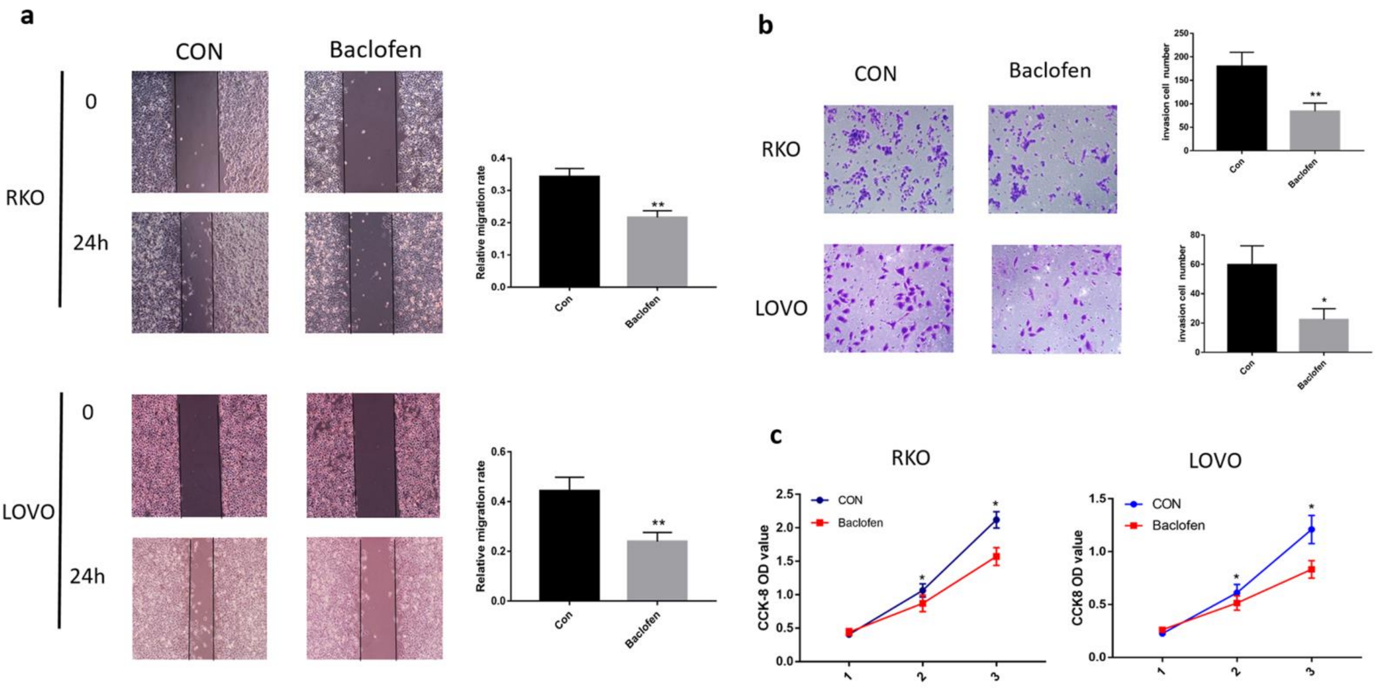

Supplementary Figure 1.

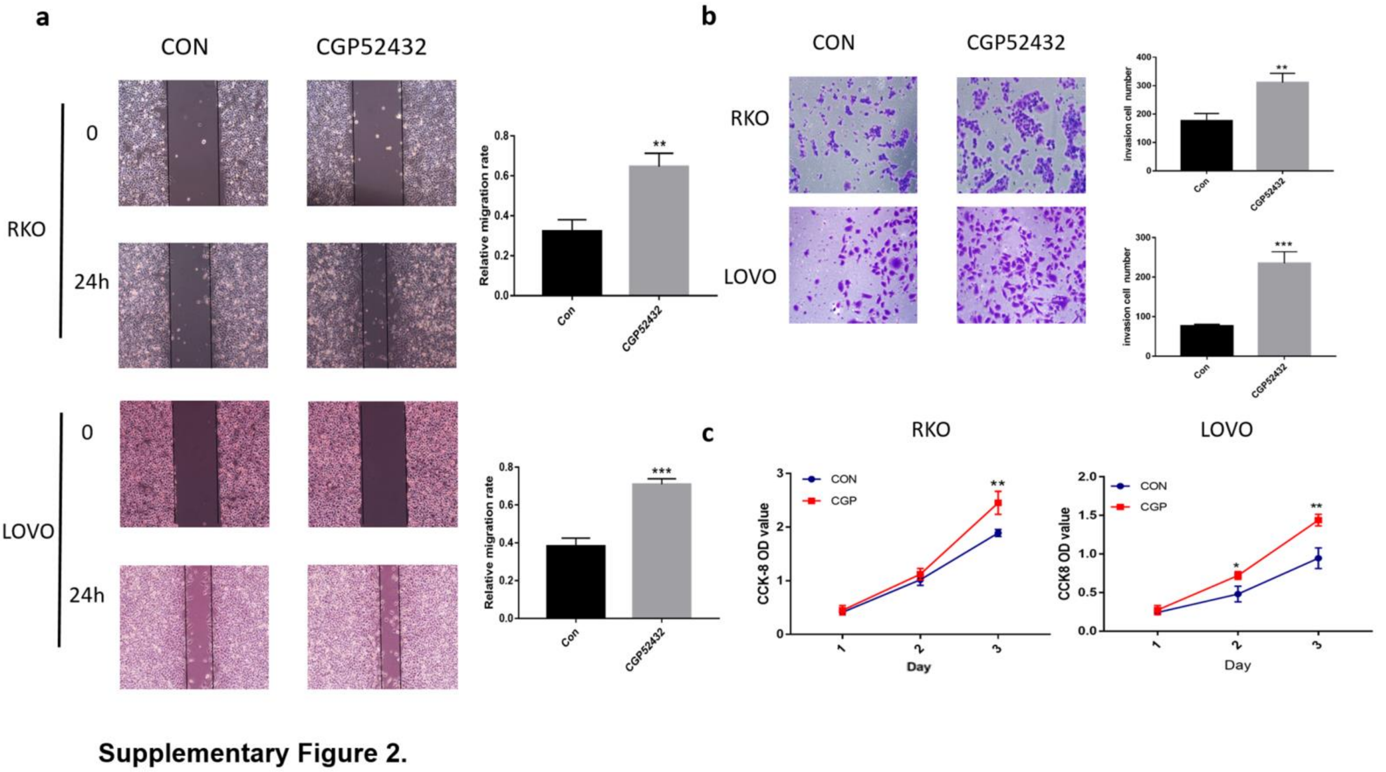

Supplementary Figure 2.
